# Proline: The Distribution, Frequency, Positioning, and Common Functional Roles of Proline and Polyproline Sequences in the Human Proteome

**DOI:** 10.1371/journal.pone.0053785

**Published:** 2013-01-25

**Authors:** Alexander A. Morgan, Edward Rubenstein

**Affiliations:** 1 Department of Biochemistry and Genome Technology Center, Stanford University Medical School, Stanford, California, United States of America; 2 Department of Medicine, Stanford University School of Medicine, Stanford, California, United States of America; Federal University of São Paulo (UNIFESP), Escola Paulista de Medicina, Brazil

## Abstract

Proline is an anomalous amino acid. Its nitrogen atom is covalently locked within a ring, thus it is the only proteinogenic amino acid with a constrained phi angle. Sequences of three consecutive prolines can fold into polyproline helices, structures that join alpha helices and beta pleats as architectural motifs in protein configuration. Triproline helices are participants in protein-protein signaling interactions. Longer spans of repeat prolines also occur, containing as many as 27 consecutive proline residues. Little is known about the frequency, positioning, and functional significance of these proline sequences. Therefore we have undertaken a systematic bioinformatics study of proline residues in proteins. We analyzed the distribution and frequency of 687,434 proline residues among 18,666 human proteins, identifying single residues, dimers, trimers, and longer repeats. Proline accounts for 6.3% of the 10,882,808 protein amino acids. Of all proline residues, 4.4% are in trimers or longer spans. We detected patterns that influence function based on proline location, spacing, and concentration. We propose a classification based on proline-rich, polyproline-rich, and proline-poor status. Whereas singlet proline residues are often found in proteins that display recurring architectural patterns, trimers or longer proline sequences tend be associated with the absence of repetitive structural motifs. Spans of 6 or more are associated with DNA/RNA processing, actin, and developmental processes. We also suggest a role for proline in Kruppel-type zinc finger protein control of DNA expression, and in the nucleation and translocation of actin by the formin complex.

## Introduction

There are about 900 naturally occurring amino acids. Evolution has selected only 21 for inclusion in the group (not distinguishing between cysteine and selenocysteine) that act as subunits in the assemblage of human proteins [1], [2]. One of these amino acids, proline, is highly anomalous in many respects; its unique features contribute to the special roles proline plays in protein structure and function. Williamson and MacArthur have previously reviewed aspects of this subject, including an analysis of proline-rich regions in some proteins [3], [4].

**Figure 1:**
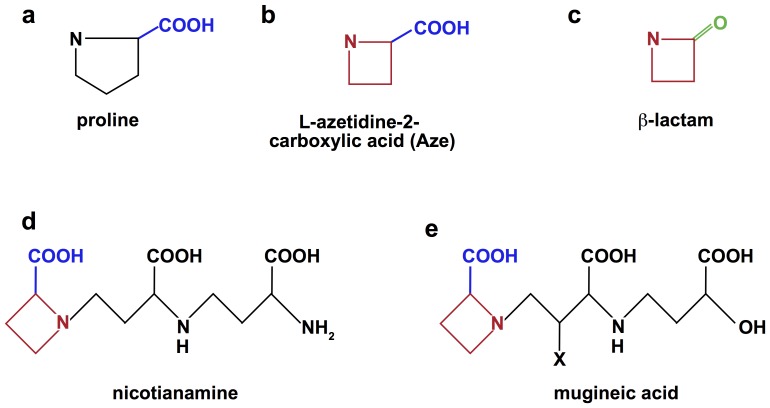
Structure of Proline and Related Homologues. This figure shows the structures of proline (a), L-azetidine-2-carboxylic acid (Aze) (b), ß-lactam (c), nicotianamine (d), and mugineic acid (e). Aze is the lower homologue of proline. Its ring has only four members instead of five. Plants synthesize Aze as essential constituents of the two metal chelating molecules, nicotianamine and mugineic acid. These compounds trap metal ions in the soil and transport them to various plant parts. Aze is in particularly high concentrations in the bulbous roots of many plants, making its way into the human and lifestock food supply. Aze exerts its toxic effects by eluding the proof-reading function of the prolyl tRNA synthetases, allowing it to be misincorporated into nascent peptides or proteins in which it replaces proline. When Aze replaces proline, it can change protein structure, function, and antigenicity. This molecular mimicry is analagous to the other 4-member nitrogen containing ring of ß-lactam, which exerts its bactericidal effects by mimicking a D-Ala-D-Ala sequence of a transpeptidase, irreversibly blocking its role in bacterial cell wall synthesis. The role of Aze in human health is yet to be established.

Proline's nitrogen atom is covalently bound within the molecule's five-membered ring, a feature that markedly restricts the phi (φ) angular range in peptide bond formation at this locus in a peptide or protein (Figure 1a). Furthermore, proline can readily adopt a *cis* configuration as well as a *trans* configuration in response to subtle influences, presumably differences in local charge distribution. This exceptional behavior accounts for the tendency of prolyls to bend the regional amino acid alignment and therefore to fold the protein [5].

Having only one hydrogen atom attached to its nitrogen, proline cannot donate protons but it can serve as a proton acceptor. Prolyls tend to be excluded from alpha helices and beta sheets. They can, however, be situated at positions at the ends of these motifs. In one simplified view, proline disrupts protein secondary structure by inhibiting the backbone to conform to an alpha-helix or beta-sheet conformation. The alternate intpretation is that proline imposes its own kind of secondary structure with a confined phi angle that overrides other forms of secondary structure. Because of their hydrophobicity they tend to adopt positions within the interior of a protein.

Tri-proline sequences may fold into right-handed or left-handed helices, referred to as polyproline I (PPI) or polyproline II (PPII), respectively [6]–[8]. The left-handed version is far more common. It forms when the consecutive residues assume approximate dihedral angles of −75° at the phi (φ) position and 150° at the psi (ψ) position and are isomerized to the *trans* position of their peptide bonds. Its left-handed helix contains three residues per turn and the rise is about 3.1 Å. On the other hand, the rarer polyproline I helix forms when the consecutive residues assume approximate dihedral angles of −75° at the φ position and 160° at the ψ position and is isomerized in the *cis* position at the peptide bond. The right-handed helix contains about 3.3 residues per turn, and the rise is only about 1.9 Å.

Polyproline helices can induce sharp turns in the local geometry. In addition to their role in molecular conformation, polyproline II helices are important in protein-protein interactions, mediating signal transduction, as with adjacent SH3, EVH1, and WW motifs [9]–[20]. Other amino acids, referred to as “guests”, such as glycine, asparagine, alanine, glutamine, valine, aspartic acid, histidine and lysine, may participate in PPII helical conformations [8], [21], [22].

Because of its unique structural properties, we were interested in determining proline's distribution across the proteome and identifying the shared functional properties of proteins with high levels of proline and long polyproline stretches. Herein we report information about the presence, distribution, and effects of proline residues and repetitive sequences in 18,666 unique human proteins. These proteins were taken from the HUGO Gene Nomenclature Committee's list of accepted protein coding genes [23]. Although the number of open reading frames transcribed into mRNA may be greater than 18,666, we believe this curated set of protein-coding genes is reasonable representation of the members of the uniquely coded human proteome. We present detailed information about the occurrence of polyproline sequences of three or more residues and their association with structure and function.

## Results

**Figure 2:**
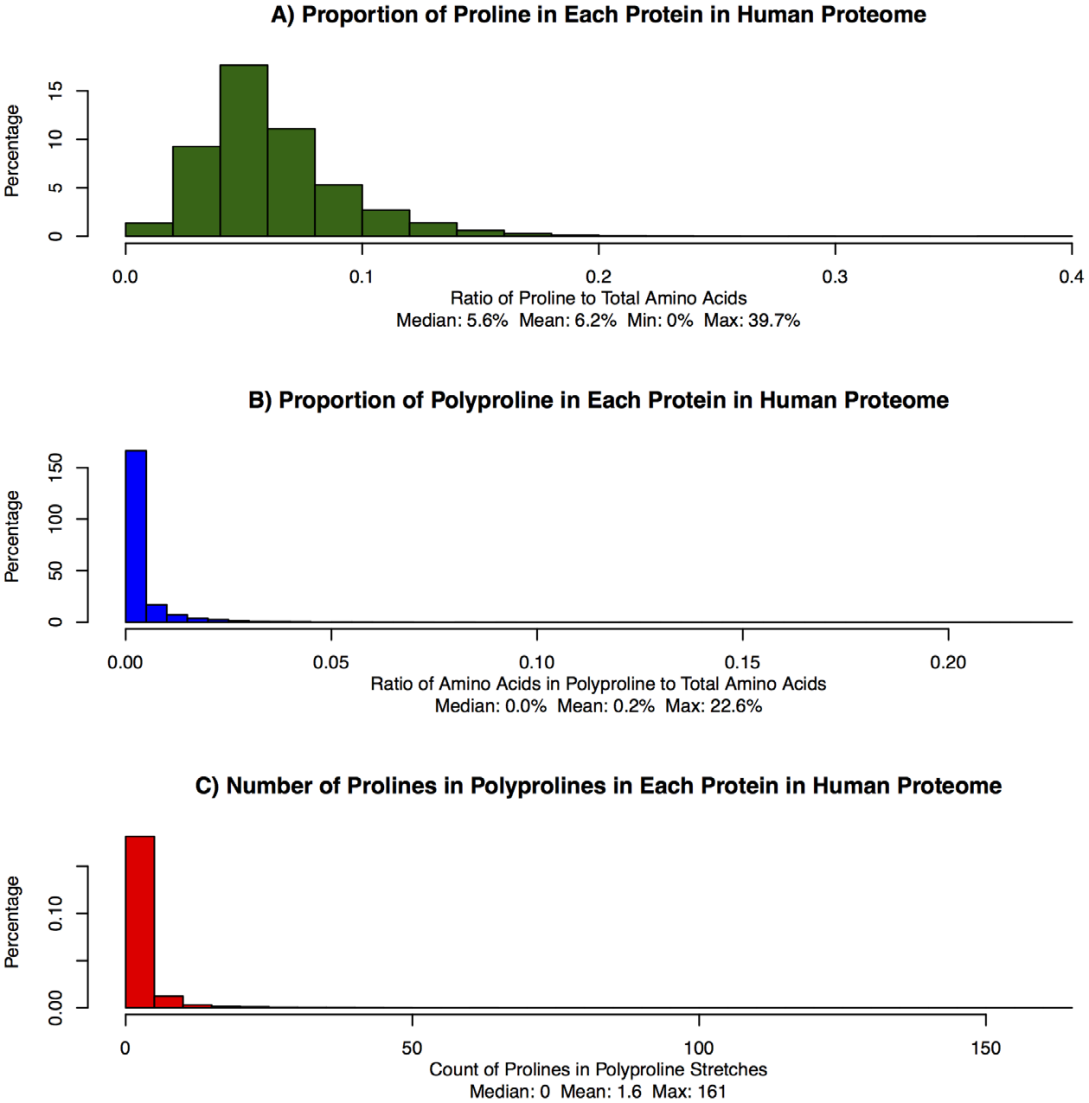
Distribution of Proline Across Proteome. (**a**) Counts of amino acids binned by their proportion of proline. (**b**) Counts of amino acids binned by their proportion of polyproline. (**c**) Counts of polyproline spans binned by their length.

**Figure 3:**
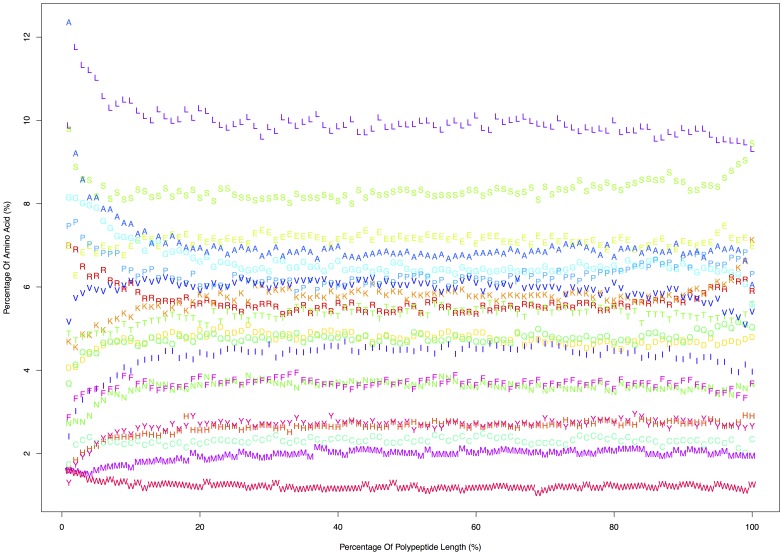
Distribution of Amino Acids by Protein Length Across Human Proteome. The counts of each amino acid as a function of relative length are shown with each letter corresponding to the appropriate amino acid. Each protein was divided into 100 segments and the total count of each amino acid in each segment was summed across the proteome. For example, a 200 amino acid long protein with a serine in position 3 and a lysine in position 4 would add a count of one S and one L in the 2% bin along the horizontal axis. Proline, P, peaks in prevalence at the 2% of length position, with 8,215 prolyl residues of a total of 108,671 amino acids (7.6% proline). The vertical axis was normalized to reflect a percent frequency.

The total number of human proteins studied was 18,666; of these, 99.8% contain proline. The median length of all the proteins is 436 residues. The proteins encompass a total of 10,882,808 amino acids, among which there are 687,434 prolyls, which account for 6.3% of all amino acids in the human proteome. The relative abundance of proline in the proteins of the human proteome is shown in the histogram in Figure 2a. The distributions of all 20 amino acids as a function of relative length are shown in the Figure 3 (Table S1). There are 564,316 singlet prolines (82.1%), 46,540 pairs of proline (13.5%), and 30,038 spans of 3 or more (4.4%).

### Proline-Rich, Polyproline-Rich, and Proline-Poor Proteins

There are 46 proteins that contain no proline (Table S2). On the other hand, the long keratinocyte envelope protein SPRR2G (small proline rich protein 2G), some 73-amino acid residues in length, is comprised of 39.7% proline. We examined the enrichment of functional annotations in the top and bottom 1% of molecules in the human proteome, as ordered by their percentage of proline, Table S3 & Table S4.

Both the proline-rich and proline-poor proteins are heavily involved with the formation of the dermis and with keratinization, but their functional roles are very different. Proline-rich proteins include the collagens (e.g. COL3A1, COL5A1, COL1A2) and proteins in the cornified envelope (e.g. LCE2C, SPR2G, LCE2B, SPRR2F) which rely on the properties of proline and hydroxy-proline to form their helices. By contrast, many of the proline-poor proteins exhibit a different coiled-coil motif that includes the intermediate filament proteins that make up the keratins (e.g.KRT9, KRT19, KRT77, KRT14). This distinction highlights the important role of proline and polyproline in determining helical structure. The two kinds of helical structures lie on opposite ends of the proline abundance scale. Proline-poor proteins and domains form one class of helices (e.g. keratins), which can assemble only by excluding proline. Proline-rich molecules, which exhibit a contrasting triple-helical conformation, such as the collagens, constitute another large group of proteins.

Many of the proline-poor proteins, such as the SNAP receptor (SNARE) complex, are involved in vesicle transport and membrane fusion, as exemplified by membrane docking, internal protein transport, and exocytosis (e.g. KDELR3, STX6, RAB2A, GNAI3, AP2S1, SNAP25). Another set of proline-poor proteins is highly enriched for the alpha-helical, calcium binding, EF-hand domain (e.g. S100A, CALB1, FLG2, MYL5). Other proline-poor proteins include the fatty acid binding family (FABP1-5) and other lipid binding proteins (e.g. PMP2, RBP2), as well as GTP binding proteins (e.g. ARL5A, RAB13, GMAI1, GTBP6).

In addition to the collagens, proline-rich proteins include some of the homeobox (HOXA3, HOXA4, ESX1, CDX1, TPRX1) and forkhead box (FOXE3, FOXN1) proteins, as well as the zinc finger proteins. The proline-rich proteins also tend to be highly enriched for consecutive sequences of prolines, so called polyproline sequences.

We examined proteins containing abundant proline, as well as proteins with stretches of contiguous prolines forming a “polyproline motif,” which we define as three or more prolines in consecutive sequence. The distribution of polyproline motifs among proteins in the human proteome is shown in Figure 2b & Figure 2c. Table S2 provides detailed information about the number of prolines in the longest spans and the number of separate polyproline sequences. Table S5 shows the start and end positions of each polyproline span.

We examined 11 proteins that start with a tri-proline repeat (ZBTB4, DHX34, HINFP, CD19, IRGQ, ELMO1, ELMO2, MAP1LC3C, IGLON5, IDS, and CPZ), along with 4 proteins that end with a tri-proline repeat (PYDC2, ARHGEF15, RHBDL1, and OR10S1). There are no sequential patterns of amino acids in any of these and there are no apparent functional commonalities among them. Protein OR10S1, which ends with a tri-proline, is an olfactory receptor protein that interacts with odorants and triggers a neuronal response. This pattern was not found in the other proteins that are believed to be odorant detectors: 52 NP olfactory receptor protein, MOR256-8, MOR256-17, MOR256-22, OMP olfactory marker protein. Overall, we could find no association between polyproline at the initial or terminal ends and protein functions.

Consecutive sequences of six or more prolines are associated with DNA/RNA processing, including zinc fingers, actin, and developmental processes. There are 27 proteins that contain from 12 to 27 consecutive repeats; there are 13 proteins with 11 repeats; there are 21 proteins with 10 repeats, and 30 proteins with 9 repeats. Their functional roles are shown in Table 1. Of the total 91 proteins in the above groups, there are 42 proteins (45%) associated with DNA/RNA processing, including 14 zinc finger proteins (15%), and 11 proteins associated with actin (12%).

**Table 1:** Association of functional classes with polyproline long repeats.

Serial Repeats	Number in Group	DNA/RNA Processing	Zinc Fingers	Actin Associated	Ubiquitin Associated
12–27	27	12	8	5	0
11	13	2	1	2	4
10	21	11	1	3	1
9	30	17	4	1	2
Totals	91	42(45%)	14(15%)	11(12%)	7(8%)

Counts of functional classes of the proteins with the longest consecutive proline spans.

### Zinc Finger Proteins

Because of the apparent over representation of zinc finger proteins, we focused on the structure and function of these molecules to gain further insight into the role of polyproline, and found that none of the first 10 display regular recurring motifs: PCLO, ZIF268, ZFP746, ZNF827, Zinc Family Member 5, Zinc Finger CCCH domain, ZFHX4, Zinc Finger Protein 318, Zinc Finger Homeobox protein 3, ZNF367, ZFP579, ZFPM1 (Figure S1). We suspect that the complex configurations introduced by polyproline helices disrupt long continuous motifs. On the other hand, acute angular changes in conformation could subserve the geometric requirements of highly articulated intra- and inter-molecular interactions.

**Figure 4:**
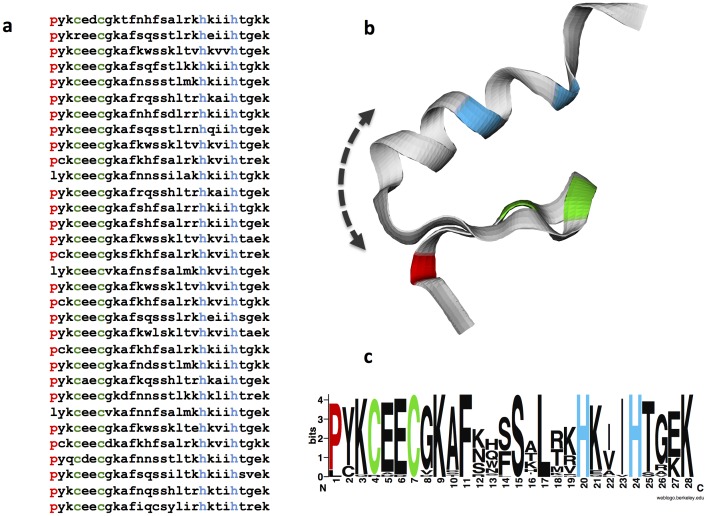
Repeated TWEAZR Motif in ZNF729. (**a**) The 28-amino-acid motif (TWEAZR) repeated 32 times in ZNF729. (**b**) Ribbon cartoon showing the likely structure of the conserved zinc finger motif based on homology with similar zinc finger structures. The cysteine residues are marked in green, the histidine in blue, and the proline in red. We propose that cis-trans isomerization of the proline can move the downstream portion of the zinc finger domain and alters the contact of some residues with specific nucleic acids. (**c**) The logo for the TWEAZR motif in ZNF729.

In some zinc finger proteins (Kruppel type) that contain only singlet or doublet prolyls (no triplets and their helices, and no longer runs of prolines), there is an amino acid motif in which a prolyl recurs every 28 residues (Figure S2). A 28-residue conserved motif is a well-known feature of some zinc finger structures [24], [25], which we call TWEAZR (Twenty-Eight Amino acid Zinc finger Repeat). It includes a linker sequence TGEH. The proline is followed by a YKCEEC sequence, and later an HXXXH sequence Figure 4. The two cysteines and the two histidines conjugate with a zinc atom. By contrast, in zinc finger proteins that contain prolyl triplets and their miniature helices, as well as longer consecutive repeats that may encompass such helices, this pattern breaks down, possibly because the small polyproline helices insert irregularities into the larger spiral contours of this class of zinc finger molecules. For instance, among the first ten proteins free of polyproline sequences (ZNF100, ZFP726, ZFP729, ZFP732, ZFP733, ZFP736, ZFP737, ZFP739, ZNF741), we found the TWEAZR motif in each, with proline recurring every 28 residues. Figure S3 shows the pattern repeated in ZNF729.

We conducted a detailed analysis of proline in zinc finger proteins according to their number of consecutive prolyl repeats, from 2 to 27. Among the 95 members containing 9 to 27 consecutive repeats, there are 13 zinc finger proteins (13.4%) (Table 2). In molecules that contain consecutive prolyl spans of three or more (highest 22), there are 4245 proteins, of which 83 are zinc finger proteins (1.95%). Among the proteins lacking repeats, there are 14,102 proteins, and 425 zinc finger proteins (0.03%). Of the first 9 zinc finger proteins that show a disorderly amino acid arrangement, 8 (89%) contain prolyl dimers in the pattern of ppx. In these 9 proteins there is a total of 63 dimers, of which 35 contain “guest” amino acids in the third position (Table S6). Common guests include glycine, asparagine, alanine, glutamine, valine, aspartic acid, histidine and lysine.

**Table 2:** Abundance of Zinc Finger Proteins with Polyproline Spans.

Prolyl Repeats	Proteins	Zinc Finger Proteins
27	1	0
22	1	1
20	3	0
15	5	2
14	2	0
13	6	0
12	9	2
11	14	1
10	21	3
9	33	4
8	47	0
7	99	4
6	147	4
5	284	5
4	530	12
3	3034	44

By contrast in the low-proline zinc finger protein group, among the first 10 proteins, there are only 5 which contain proline dimers, all with guests in the third position (Table S7). In the total there are 50 zinc finger proteins in this subgroup, containing a total of 659,569 amino acids, and 28 prolyl dimers. Prolyl dimers account for only 0.00004% of the residues. Thus prolyl dimers are rare in such zinc finger proteins.

The repetitive pattern, TWEAZR, with prolyls recurring every 28 residues, was not found in any of the 10 zinc finger proteins containing the longest consecutive spans or in proteins with the highest percentage of prolyl residues (20%). To determine whether one or more proline trimers in a molecule is associated with the presence or absence of TWEAZR, we compared the frequency of such patterns among zinc finger proteins in which there are one or more trimers, with the frequency of such patterns in molecules in which there are no trimers. There are 43 zinc finger protein molecules in the first group. Of these, only two (ZNF189 and ZNF283) display the repetitive motif, or a frequency of 4.6%. Noteworthy is the fact that in both cases the ppp triplet is located near the beginning of the lead sequence. In ZNF189 (612 amino acids) the triplet prolyls occur in positions 6,7, and 8. In ZNF 283 (679 amino acids) the prolyl triplet occupies positions 22, 23, and 24. On the other hand, of the first 43 zinc finger proteins in the “0” category in which the molecules contain no consecutive prolyl spans beyond 2 (that is, dimers), there are 32 molecules that display the recurring TWEAZR motif (74%), and 11 molecules (26%) that lack it.

These results indicate that a polyproline sequence of three is unlikely to be associated with the presence of the recurring TWEAZR motif within a zinc finger molecule. In the unusual instances in which tri-prolines are present, they appear to be limited to the lead sequence and do not occur within the repetitive domains.

It is suggested that tri-proline helices disrupt the repetitive amino acid zinc finger protein sequences that we have noted and that have been previously described [26], [27]. This conclusion is supported by that fact that of the 144 proteins in which there are 8 to 27 polyproline repeats, only formin-2 (NP_064450.3), a 1,722 amino acid actin-associated protein, displays a repetitive motif. This consists of 22 consecutive sequences comprised of a quintet of prolines, each followed by *lpgagi*, commencing at residue number 976 and ending at residue number 1211 (Figure S4). Formins are multidomain proteins that are involved in actin nucleation [28], [29].

### Genetic and Acquired Disorders Related to Proline

There is a voluminous literature about hereditary disease caused by mutations involving proline. PubMed lists 6,068 citations, as of 26 May 2012. Little is known about acquired disease in humans caused by the ingestion of azetidine-2-carboxylic acid (Aze), the lower homologue of proline, containing four members in its ring instead of five (Figure 1). It is a constituent of the diet. Aze eludes the gatekeeping function of prolyl aminoacyl tRNA synthetases, and is misincorporated into proteins in place of proline [1], [30], [31].

## Discussion

Gene processing proteins, such as those proline rich proteins that catalyze splicing of a primary gene transcript (pre-mRNA), lead to translation of a single message into a large number of different protein isoforms. Thus the misassembly of one alternatively splicing protein can result in the malformation of a very large number of downstream protein products. The result is the marked amplification of the effects of a single protein misconstruction. Such events during early embryogenesis could have damaging consequences. They may also contribute to carcinogenesis.

Zinc finger proteins are intimately involved in DNA expression, RNA assembly, transcription, and apoptosis [32], [33]. Our data indicate that in one class of zinc finger proteins that contain only singlet or doublet prolyls – no triplets and their helices or longer runs of prolines, there is an amino acid motif (TWEAZR) in which a prolyl recurs every 28 residues and very rarely anywhere else. It follows the sequence TGEH, and preceeds a YKCEEC sequence Figure 4. Why evolution has selected proline for this specific position in these molecules is yet to be determined. Obviously the size, shape, charge distribution, and peptide angles are important. Beyond these properties is the fact that proline readily isomerizes between the *trans* and *cis* positions. Its flexing action shifts the location of variable residues downstream, as in positions 13 and 16. These residues in ZNF 729 are shown in Table 3, in which we arbitrarily assigned proline to residue position 1. In the 30-sequence repeats, position 14 is occupied by a serine 18 times and by a phenylalanine 12 times. Position 15 is always occupied by a serine. In zinc finger proteins 100, 141, 208, 726, and 737, position 13 is occupied by 15 different amino acids, and position 16 by 12 different amino acids. On the other hand, position 14 is occupied by serine and phenylalanine 56% and 33% of the time, respectively. Position 15 is occupied by serine 97% of the time. These molecular locations may be relevant to zinc finger amino acid/DNA contact [34]. In ZNF 729 the 30 residues in position 13 and in position 16, 24 are polar and 6 are nonpolar. Of the 30 residues in position 14, 18 are polar and 12 are nonpolar, and all 30 residues in position 15 are polar.

**Table 3:** Conservation and variation in the amino acids in the repeating motif in ZNF729.

Position	Number of conserved amino acids	Number of varying amino acids
1	29 p	3 l
2	27 y	5 c
3	31 k	5 c
4	31 c	1 r
5	30 e	1 a, 1 d
6	31 e	1 d
7	32 c	
8	31 g	1 v
9	32 k	
10	31 a	1 t
11	32 f	
12	12 k	10 n, 7 s, 2 r, 1 i
13	10 q	9 h, 6 w, 4 n, 2 s, 1 d,
14	18 s	12 f, 1 l, 1 c
15	32 s	
16	10 a	7 t, 6 k, 4 h, 2 i,1 d, 1 y, 1 s
17	32 l	
18	12 t	12 r, 4 m, 2 k, 1 a, 1 i
19	17 k	8 r, 5 v, 1 n, 1 e
20	32 h	
21	29 k	2 e, 1 q
22	15 i	11 v, 3 a, 2 t, 1 l
23	31 i	1 v
24	32 h	
25	31 t	1 s
26	25 g	2 a, 4 r, 1 v
27	23 e	9 k
28	32 k	

Perhaps some of these variable amino acids make on-off contact with bases in DNA, depending on the *cis/trans* state of the preceding proline. Thus prolyl isomerization may be a highly conserved mechanism that determines zinc finger protein control of DNA expression. This pivotal role of proline is supported by the fact that other prolyls are virtually excluded within the recurring motif domain. We found only 3 prolines in 2,084 outlying positions, a frequency of 1∶695. The isomerization of an outlier could corrupt the logic of the molecule's structure. This hypothesis about the uniqueness of proline in this setting is strengthened by the fact that single residues of each of the other amino acids that form the repetitive sequences (t, g, a, e, k, y, c, and h) are found in random locations elsewhere within the molecule.

In addition, the critical role of proline isomerization in zinc finger function is supported by the close association between zinc finger proteins and proteins that possess peptidyl-prolyl isomerase activity, such as cyclophilin, FKBP's, and parvulin [35]. Cyclophilin A, a peptidyl-prolyl isomerase, has been shown to be necessary for the proper function of the zinc finger protein Zpr1p [36]. We suggest that the control of prolyl isomerization may be a critical link in zinc finger protein regulation of gene expression.

Furthermore, in zinc finger proteins that contain long consecutive prolyl repeats, including prolyl triplets and their miniature helices, this cyclic pattern breaks down. Presumably the polyproline helices introduce abrupt changes in the direction of downstream residues, and interfere with the formation of long continuous spiral domains. Curiously, zinc finger proteins are markedly over represented among these proline-laden molecules. It is apparent that zinc finger protein structure and function may be corrupted by the substitution of Aze for proline.

Overall, we have cataloged the presence, position, and general functional role of proline in the human proteome. We have suggested a possible role for proline in the regulation of zinc finger protein binding to nucleic acids based on *cis-trans* isomerization. Also, by listing the dominant functional roles of proline-rich proteins, we suggest likely future directions for the investigation of the impact of Aze misincorporation on human molecular pathophysiology. Such studies have been pioneered by Schimmel and his colleagues [37].

## Methods

To define the human proteome, we started with the human genome. Using the HUGO Gene Nomenclature Committee's list of accepted protein coding genes [23], we obtained the corresponding protein sequences for these genes from Ensembl [38]. For each gene, we used the longest peptide sequence in Ensembl corresponding to each gene and removed the initial methionine in each case, giving us 18,666 total different amino acid sequences making up the human proteome.

Amino acid statistics were computed using the R statistical programming language [39]. We examined functional enrichment of lists of proteins rich or poor in proline or polyproline using the web-based DAVID tool from NIAID [40].

## Supporting Information

Figure S1 **Five Examples of High Proline-Containing Zinc Finger Proteins Lacking a Repetitive Motif (TEAZR).** The amino sequences of five proteins high in proline content which lack the zinc finger motif TEAZR. Proline is highlighted in bold in these sequences.
(TIFF)

Figure S2 **Zinc Finger Proteins of the Kruppel-type.** These are examples of zinc finger proteins of the Kruppel type. They display a repetitive 28-residue structural motif. These proteins are free of consecutive prolyl repeats beyond dimers.
(TIFF)

Figure S3 **Amino Acid Sequence of ZNF729.** Prolines are highlighted in bold in this sequence of ZNF729.
(TIFF)

Figure S4 **Amino Acid Sequence of Human Formin.** Prolines are highlighted in bold in this sequence of Formin.
(PDF)

Table S1 **Counts of Amino Acids by Protein Length Across Human Proteome.** The counts of each amino acid as a function of relative length are shown. Each protein was divided into 100 segments and the total count of each amino acid in each segment was summed across the proteome. For example, a 200 amino acid long protein with a serine in position 3 and a lysine in position 4 would add a count of one S and one L in the 2% bin along the horizontal axis. Proline peaks in prevalence at the 2% of length position, with 8,215 prolyl residues of a total of 108,671 amino acids (7.6% proline).
(XLS)

Table S2 **Distribution of Proline and Polyproline Across the Human Proteome.** This table compiles the counts of proline and polyproline spans by gene. The first column is the gene symbol, followed by the length of the longest span of proline in the protein (*MaxSpan*). *NumberPolyproline* indicates the number of spans of continuous proline of 3 or more members. *PeptideLength* is the overall protein length in residues. *PolyprolineValues* is a shorthand summary of the appearance of spans of contiguous proline; for example, “PPPP:207:210 PPP:399:401” indicates that there are four prolines at positions 207–210, and another three at positions 399–401. There is a column for each canonical amino acid, with the counts for each in the protein. The *P* column shows the total number of prolyl residues in the protein. *PinPolyproline* and *PNotPolyproline* give the number of proline in polyproline spans and not in polyproline spans, respectively. *LeftStart* indicates the position of the start of the first polyproline span, whereas *LeftStartRatio* scales this by the total protein length. *RightEnd* and *RightEndRatio* indicate the end position of the final polyproline in the peptide. *SingletCount* is the number of single prolines, and *DimerCount* is the count of non-overlapping dyads of the prolyl residues.
(XLS)

Table S3 **Enrichment of Functional Annotations for Proline-Rich Proteins.** This table shows the functional and sequence annotations derived from DAVID for the proline-rich genes (top 1% of proline content in the proteome). The polyproline rich genes are similar in functional enrichment.
(XLS)

Table S4 **Enrichment of Functional Annotations for Proline-Poor Proteins.** This table shows the functional and sequence annotations derived from DAVID for the proline-poor genes (bottom 1% of proline content in the proteome).
(XLS)

Table S5 **Start and Stop Positions of Polyproline Motifs Across Human Proteome.** The start and stop positions of each polyproline span in the human proteome is shown. There are duplicate gene symbols for proteins with multiple polyproline spans.
(XLS)

Table S6 **Prolyl dimers and associated guests in zinc finger proteins.** The first nine zinc finger proteins that show disorderly amino acid sequences.
(TIFF)

Table S7 **Zinc finger proteins with low proline occurrence.** The proline dimers in zinc finger proteins lacking three or more consecutive prolyl residues. All contained guests in the third position, implying that they may take on a polyproline helical configuration. In the above group most of the prolyl dimers are located in the lead sequence, at a considerable distance from the repeat motifs.
(TIFF)

## References

[1] Rubenstein E (2000) Biologic effects of and clinical disorders caused by nonprotein amino acids. Medicine 79: 80–89. 10771706 10.1097/00005792-200003000-00002

[2] Bell EA (2003) Nonprotein amino acids of plants: significance in medicine, nutrition, and agriculture. Journal of agricultural and food chemistry 51: 2854–2865. 12720365 10.1021/jf020880w

[3] Williamson MP (1994) The structure and function of proline-rich regions in proteins. The Biochemical journal 297 Pt 2: 249–260. 8297327 10.1042/bj2970249 PMC1137821

[4] MacArthur MW, Thornton JM (1991) Influence of proline residues on protein conformation. Journal of molecular biology 218: 397–412. 2010917 10.1016/0022-2836(91)90721-h

[5] Baldwin RL (2008) The search for folding intermediates and the mechanism of protein folding. Annu Rev Biophys 37: 1–21. 18573070 10.1146/annurev.biophys.37.032807.125948

[6] Adzhubei AA, Sternberg MJ (1993) Left-handed polyproline II helices commonly occur in globular proteins. Journal of molecular biology 229: 472–493. 8429558 10.1006/jmbi.1993.1047

[7] Avbelj F, Baldwin RL (2009) Origin of the change in solvation enthalpy of the peptide group when neighboring peptide groups are added. Proc Natl Acad Sci U S A 106: 3137–3141. 19202077 10.1073/pnas.0813018106 PMC2651266

[8] Mansiaux Y, Joseph AP, Gelly JC, de Brevern AG (2011) Assignment of PolyProline II conformation and analysis of sequence–structure relationship. PLoS One 6: e18401. 21483785 10.1371/journal.pone.0018401 PMC3069088

[9] Gushchina LV, Gabdulkhakov AG, Nikonov SV, Filimonov VV (2011) High-resolution crystal structure of spectrin SH3 domain fused with a proline-rich peptide. Journal of biomolecular structure & dynamics 29: 485–495. 22066535 10.1080/07391102.2011.10507400

[10] McDonald CB, Seldeen KL, Deegan BJ, Farooq A (2009) SH3 domains of Grb2 adaptor bind to PXœàPXR motifs within the Sos1 nucleotide exchange factor in a discriminate manner. Biochemistry 48: 4074–4085. 19323566 10.1021/bi802291y PMC2710136

[11] Peterson FC, Volkman BF (2009) Diversity of polyproline recognition by EVH1 domains. Frontiers in bioscience : a journal and virtual library 14: 833–846. 10.2741/3281 PMC3882067 19273103

[12] Hodonsky CJ, Kleinbrink EL, Charney KN, Prasad M, Bessling SL, et al (2012) SOX10 regulates expression of the SH3-domain kinase binding protein 1 (Sh3kbp1) locus in Schwann cells via an alternative promoter. Molecular and cellular neurosciences 49: 85–96. 22037207 10.1016/j.mcn.2011.10.004 PMC3277675

[13] Kemper B (2004) Structural basis for the role in protein folding of conserved proline-rich regions in cytochromes P450. Toxicology and applied pharmacology 199: 305–315. 15364546 10.1016/j.taap.2003.11.030

[14] Rath A, Davidson AR, Deber CM (2005) The structure of “unstructured” regions in peptides and proteins: role of the polyproline II helix in protein folding and recognition. Biopolymers 80: 179–185. 15700296 10.1002/bip.20227

[15] Kaneko T, Li L, Li SS (2008) The SH3 domain–a family of versatile peptide- and protein-recognition module. Frontiers in bioscience : a journal and virtual library 13: 4938–4952. 18508559 10.2741/3053

[16] Harauz G, Ladizhansky V, Boggs JM (2009) Structural polymorphism and multifunctionality of myelin basic protein. Biochemistry 48: 8094–8104. 19642704 10.1021/bi901005f

[17] Martin-Garcia JM, Ruiz-Sanz J, Luque I (2012) Interfacial water molecules in SH3 interactions: a revised paradigm for polyproline recognition. The Biochemical journal 442: 443–451. 22115123 10.1042/BJ20111089

[18] Nomme J, Fanning AS, Caffrey M, Lye MF, Anderson JM, et al (2011) The Src homology 3 domain is required for junctional adhesion molecule binding to the third PDZ domain of the scaffolding protein ZO-1. The Journal of biological chemistry 286: 43352–43360. 22030391 10.1074/jbc.M111.304089 PMC3234847

[19] Smith FJ, Del Monaco M, Steijlen PM, Munro CS, Morvay M, et al (1999) Novel proline substitution mutations in keratin 16 in two cases of pachyonychia congenita type 1. The British journal of dermatology 141: 1010–1016. 10606845 10.1046/j.1365-2133.1999.03198.x

[20] Stapley BJ, Creamer TP (1999) A survey of left-handed polyproline II helices. Protein science : a publication of the Protein Society 8: 587–595. 10091661 10.1110/ps.8.3.587 PMC2144280

[21] Kelly MA, Chellgren BW, Rucker AL, Troutman JM, Fried MG, et al (2001) Host-guest study of left-handed polyproline II helix formation. Biochemistry 40: 14376–14383. 11724549 10.1021/bi011043a

[22] Moradi M, Babin V, Sagui C, Roland C (2011) PPII propensity of multiple-guest amino acids in a proline-rich environment. The journal of physical chemistry B 115: 8645–8656. 21630640 10.1021/jp203874f

[23] Seal RL, Gordon SM, Lush MJ, Wright MW, Bruford EA (2011) genenames.org: the HGNC resources in 2011. Nucleic Acids Res 39: D514–519. 20929869 10.1093/nar/gkq892 PMC3013772

[24] Looman C, Abrink M, Mark C, Hellman L (2002) KRAB zinc finger proteins: an analysis of the molecular mechanisms governing their increase in numbers and complexity during evolution. Molecular biology and evolution 19: 2118–2130. 12446804 10.1093/oxfordjournals.molbev.a004037

[25] Klug A (2010) The discovery of zinc fingers and their development for practical applications in gene regulation and genome manipulation. Quarterly reviews of biophysics 43: 1–21. 20478078 10.1017/S0033583510000089

[26] Emerson RO, Thomas JH (2009) Adaptive evolution in zinc finger transcription factors. PLoS Genet 5: e1000325. 19119423 10.1371/journal.pgen.1000325 PMC2604467

[27] Kaczynski J, Cook T, Urrutia R (2003) Sp1- and Kruppel-like transcription factors. Genome biology 4: 206. 12620113 10.1186/gb-2003-4-2-206 PMC151296

[28] Courtemanche N, Pollard TD (2012) Determinants of Formin Homology 1 (FH1) domain function in actin filament elongation by formins. The Journal of biological chemistry 287: 7812–7820. 22247555 10.1074/jbc.M111.322958 PMC3293521

[29] Vizcarra CL, Kreutz B, Rodal AA, Toms AV, Lu J, et al (2011) Structure and function of the interacting domains of Spire and Fmn-family formins. Proc Natl Acad Sci U S A 108: 11884–11889. 21730168 10.1073/pnas.1105703108 PMC3141961

[30] Rubenstein E, McLaughlin T, Winant RC, Sanchez A, Eckart M, et al (2009) Azetidine-2-carboxylic acid in the food chain. Phytochemistry 70: 100–104. 19101705 10.1016/j.phytochem.2008.11.007

[31] Bessonov K, Vassall KA, Harauz G (2012) Parameterization of the proline analogue Aze (azetidine-2-carboxylic acid) for molecular dynamics simulations and evaluation of its effect on homo-pentapeptide conformations. Journal of molecular graphics & modelling 39C: 118–125. 10.1016/j.jmgm.2012.11.006 23261881

[32] Beckerle MC (1997) Zyxin: zinc fingers at sites of cell adhesion. BioEssays : news and reviews in molecular, cellular and developmental biology 19: 949–957. 10.1002/bies.950191104 9394617

[33] Laity JH, Lee BM, Wright PE (2001) Zinc finger proteins: new insights into structural and functional diversity. Current opinion in structural biology 11: 39–46. 11179890 10.1016/s0959-440x(00)00167-6

[34] Stoll R, Lee BM, Debler EW, Laity JH, Wilson IA, et al (2007) Structure of the Wilms tumor suppressor protein zinc finger domain bound to DNA. Journal of molecular biology 372: 1227–1245. 17716689 10.1016/j.jmb.2007.07.017

[35] Wang P, Heitman J (2005) The cyclophilins. Genome biology 6: 226. 15998457 10.1186/gb-2005-6-7-226 PMC1175980

[36] Lu KP, Finn G, Lee TH, Nicholson LK (2007) Prolyl cis-trans isomerization as a molecular timer. Nature chemical biology 3: 619–629. 17876319 10.1038/nchembio.2007.35

[37] Park SG, Schimmel P, Kim S (2008) Aminoacyl tRNA synthetases and their connections to disease. Proc Natl Acad Sci U S A 105: 11043–11049. 18682559 10.1073/pnas.0802862105 PMC2516211

[38] Kinsella RJ, Kahari A, Haider S, Zamora J, Proctor G, et al (2011) Ensembl BioMarts: a hub for data retrieval across taxonomic space. Database : the journal of biological databases and curation 2011: bar030. 21785142 10.1093/database/bar030 PMC3170168

[39] Ihaka R, Gentleman R (1996) R: A Language for Data Analysis and Graphics. Journal of Computational and Graphical Statistics 5: 299–314.

[40] Dennis G Jr, Sherman BT, Hosack DA, Yang J, Gao W, et al (2003) DAVID: Database for Annotation, Visualization, and Integrated Discovery. Genome biology 4: P3. 12734009 PMC193660

