# Supplementary figures and images for: Proline: The Distribution, Frequency, Positioning, and Common Functional Roles of Proline and Polyproline Sequences in the Human Proteome
Source: PLoS One. 2013 Jan 25;8(1):e53785. doi: 10.1371/journal.pone.0053785 (PMC3556072; doi:10.1371/journal.pone.0053785)

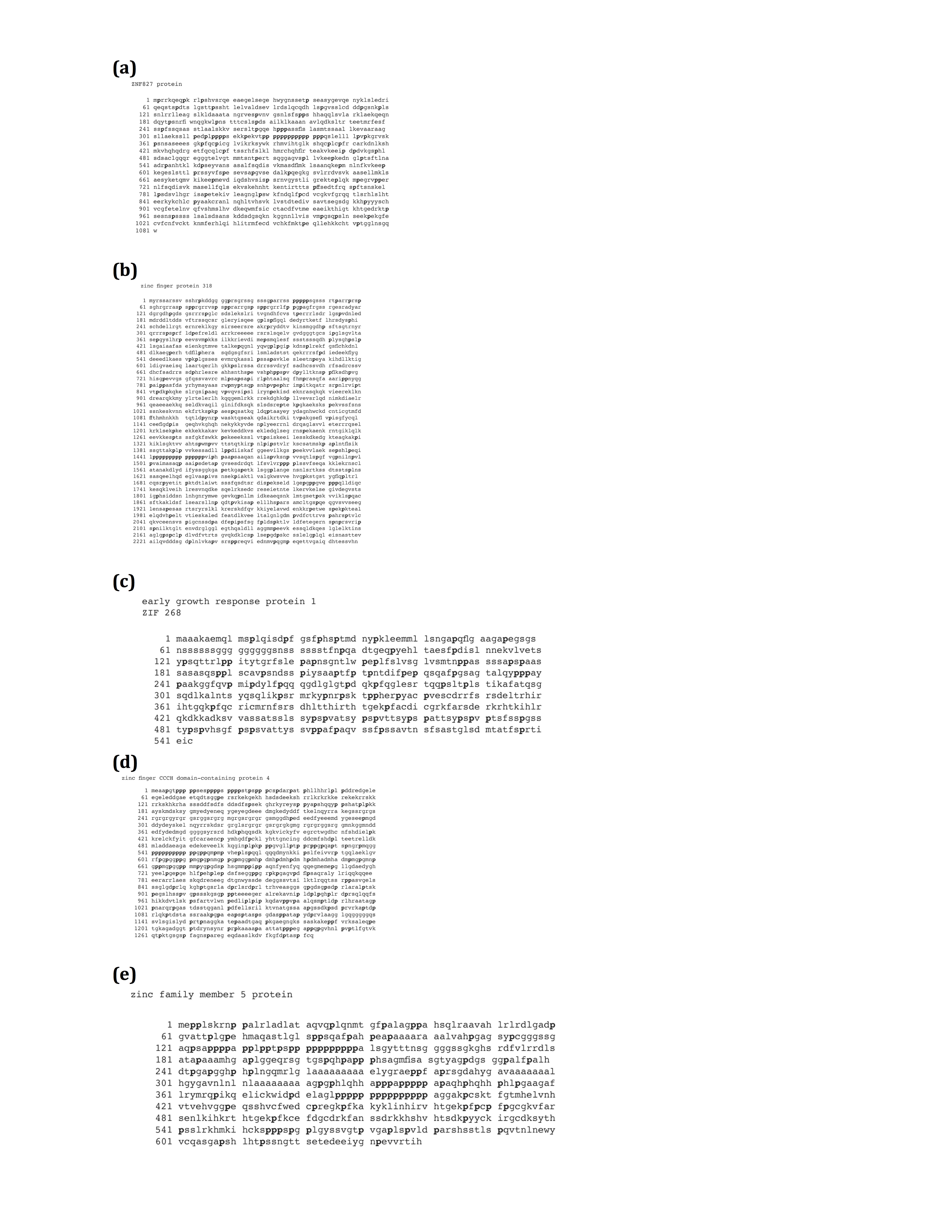

Supplement: Figure S1 — Five Examples of High Proline-Containing Zinc Finger Proteins Lacking a Repetitive Motif (TEAZR). The amino sequences of five proteins high in proline content which lack the zinc finger motif TEAZR. Proline is highlighted in bold in these sequences. (TIFF) [file pone.0053785.s001.tiff]

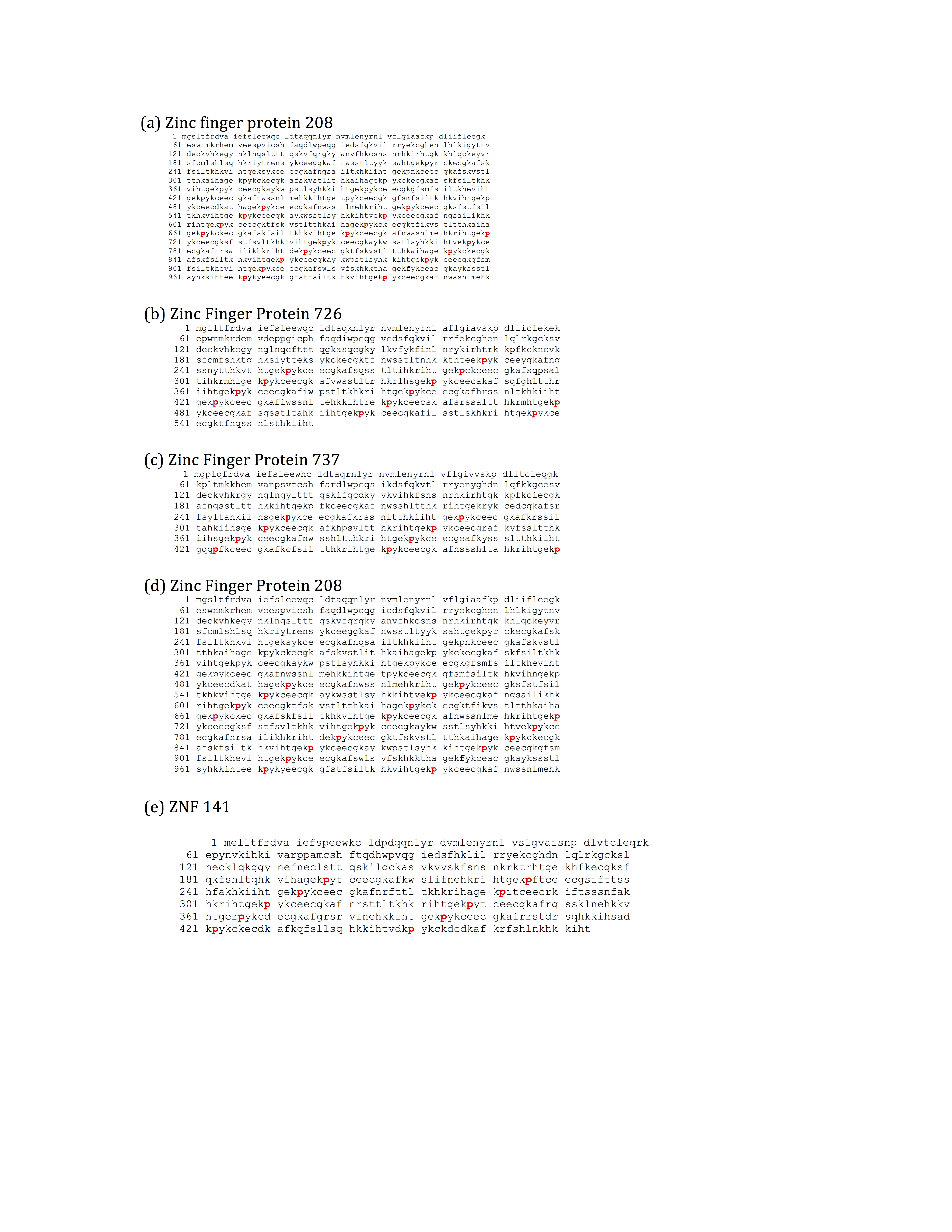

Supplement: Figure S2 — Zinc Finger Proteins of the Kruppel-type. These are examples of zinc finger proteins of the Kruppel type. They display a repetitive 28-residue structural motif. These proteins are free of consecutive prolyl repeats beyond dimers. (TIFF) [file pone.0053785.s002.tiff]

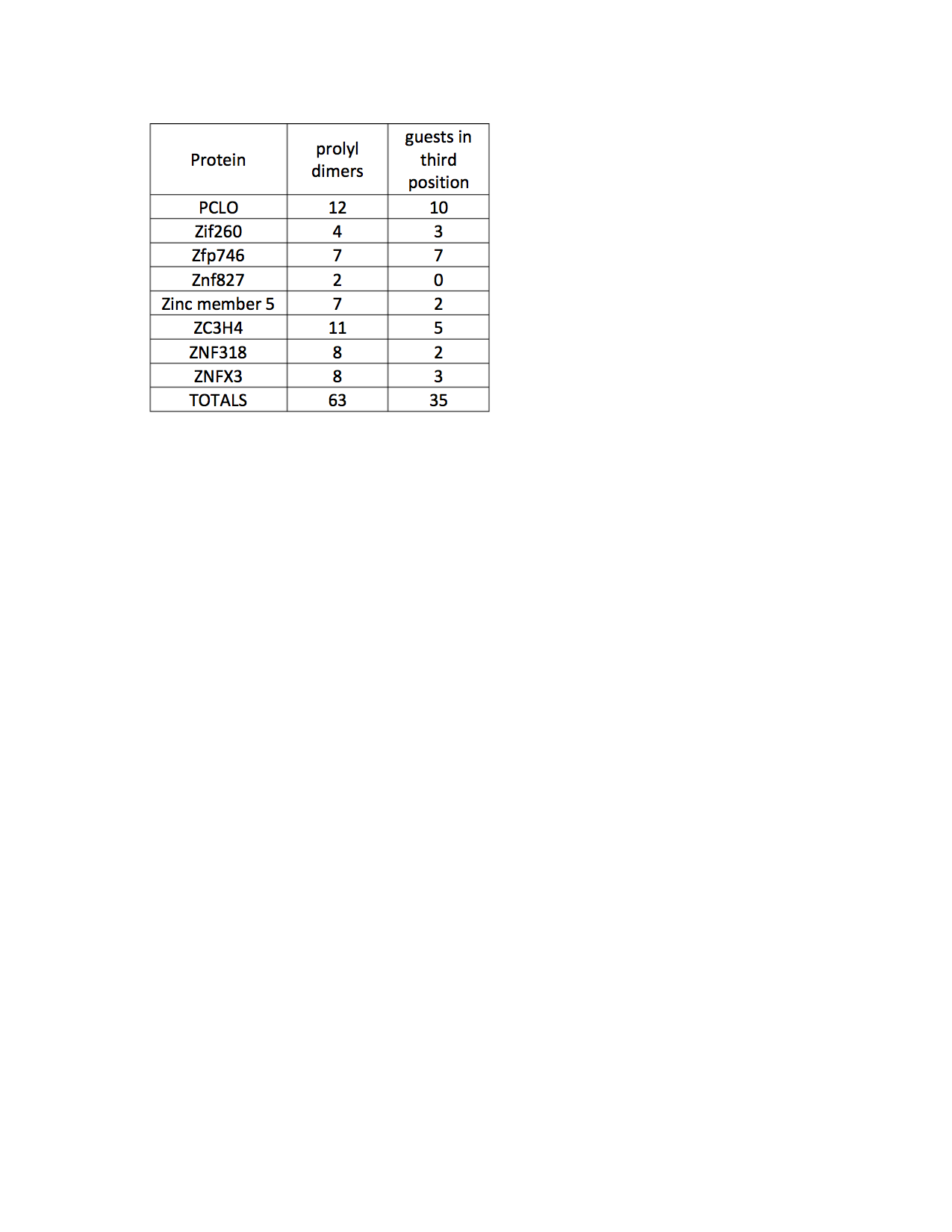

Supplement: Table S6 — Prolyl dimers and associated guests in zinc finger proteins. The first nine zinc finger proteins that show disorderly amino acid sequences. (TIFF) [file pone.0053785.s010.tiff]

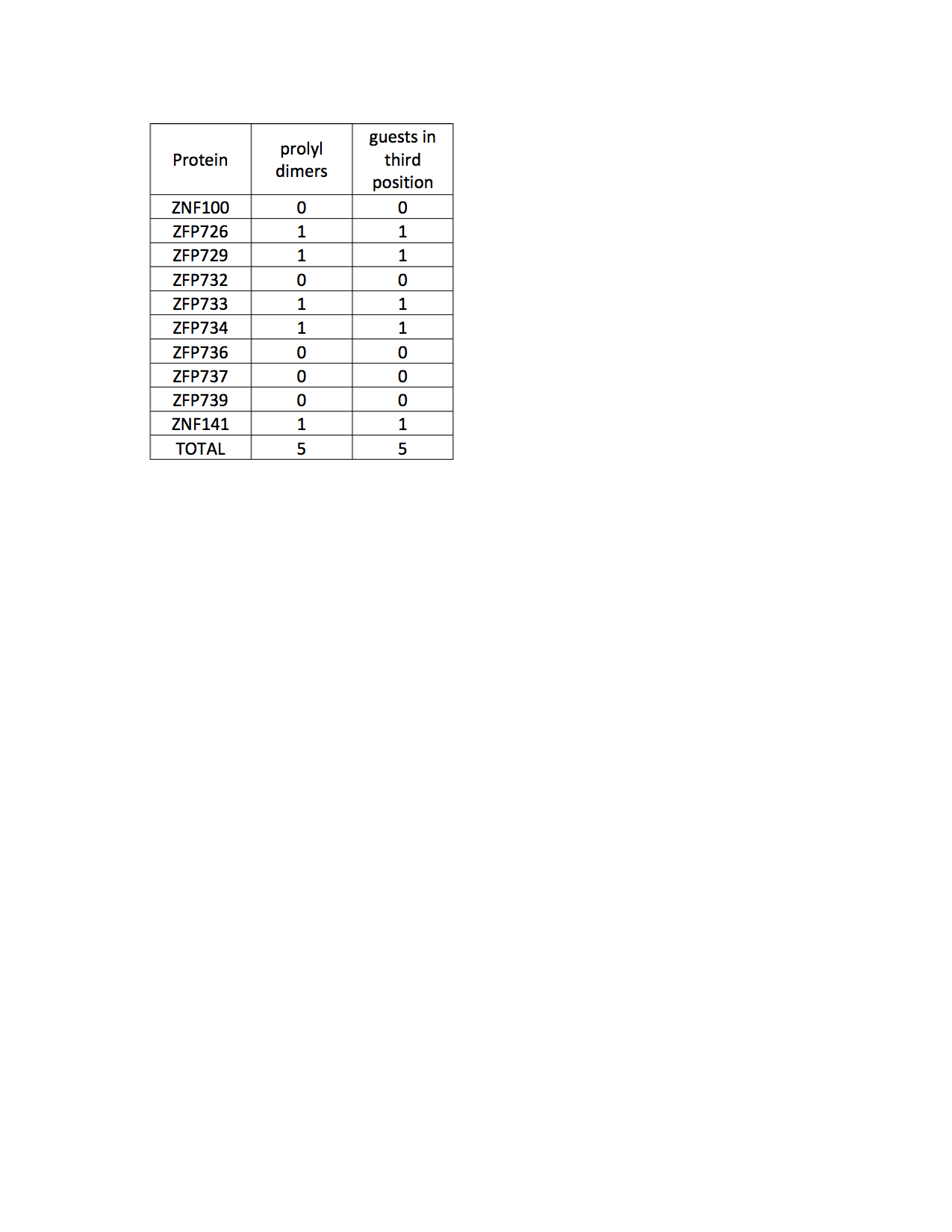

Supplement: Table S7 — Zinc finger proteins with low proline occurrence. The proline dimers in zinc finger proteins lacking three or more consecutive prolyl residues. All contained guests in the third position, implying that they may take on a polyproline helical configuration. In the above group most of the prolyl dimers are located in the lead sequence, at a considerable distance from the repeat motifs. (TIFF) [file pone.0053785.s011.tiff]
