# Supplementary material for: Proline: The Distribution, Frequency, Positioning, and Common Functional Roles of Proline and Polyproline Sequences in the Human Proteome
Source: PLoS One. 2013 Jan 25;8(1):e53785. doi: 10.1371/journal.pone.0053785 (PMC3556072; doi:10.1371/journal.pone.0053785)
Supplement: Figure S4 — Amino Acid Sequence of Human Formin. Prolines are highlighted in bold in this sequence of Formin. (PDF) [file pone.0053785.s004.pdf]

Supplementary Figure 4: Amino Acid Sequence of Human Formin

Prolines are highlighted in bold in this sequence of Formin.

```
formin 2 [Homo sapiens]

   1 mgnqdgklkr sagdalhegg ggaedalgpr dveatkkgsg gkkalgkhgk ggggggggge
  61 sgkkksksds rasvfsnlri rknlskgkga ggsredvlds qalqtgelds ahslltktpd
 121 lslsadeagl sdtecadpfe vtgpggpgpa earvggrpia edvetaagaq dgqrtssgsd
 181 tdiysfhsat eqedllsdiq qairlqqqqq qqlqlqlqqq qqqqlqgae epaapptavs
 241 pqpgaflgld rfllgpsgga geapgspdte qalsalsdlp eslaaeprep qqppspgglp
 301 vseapslpaa qpaakdspss tafpfpeagp geeaagapvr gagdtdeege edafedaprg
 361 spgeewapev gedapqrlge epeeeaqgpd apaaaslpgs papsqrcfkp yplitpcyik
 421 tttrqlsspn hspsqspnqs prikrrpeps lsrgsrtala svaapakkhr adgglaagls
 481 rsadwteelg artprvggsa hllergvasd sgggvspala akasgapaaa dgfqnvftgr
 541 tlleklfsqq engppeeaek fcsriiamgl llpfsdcfre pcnqnaqtna asfdqdqlyt
 601 waavsqpths ldysegqfpr rvpsmgppsk ppdeehrled aetesqsavs etpqkrsdav
 661 qkevvdmkse gqatviqqle qtiedlrtki aelerqypal dtevasghqg lengvtasgd
 721 vclealrlee kevrhhrile aksiqtspte eggvltlppv dglVgrppcp pgaesgpqtk
 781 fcseislivs prrisvqlds hqptqsisqp ppppsllwsa gqgqpgsqpp hsistefqts
 841 hehsvssafk nscnipsppp lpctessssm pglgmvpppp pplpgmtvpt lpstaipqpp
 901 plqgtemlpp pppplpgagi ppppplpgag ilplpplpga gipppppplpg aaipppppplp
 961 gagiplpppl pgagippppp lpgagipppp plpgagippp pplpgagipp ppplpgagip
1021 pppplpgagi ppppplpgag ipppppplpga gipppppplpg agipppppplp gagippppppl
1081 pgagippppp lpgagipppp plpgvgippp pplpgagipp ppplpgagip pppplpgagi
1141 ppppplprvg ipppppplpga gipppppplpg agipppppplp gvgippppppl pgvgippppp
1201 lpgagipppp plpgmgippa papplpppgt gipppplllpv sgppllpqvg sstlptpqvc
1261 gflppplpsg lfglgmnqdk gsrkqpiepc rpmkplywtr iqlhskrdss tsliwekiee
1321 psidchefee lfsktavker kkpisdtisk tkakqvvkll snkrsqavgi lmsslhldmk
1381 diqhavvnld nsvvdletlq alyenraqsd elekiekhgr sskdkenaks ldkpeqflye
1441 lslipnfser vfcilfqstf sesicsirrk lellqklcet lkngpgvmqv lglvlafgny
1501 mnggnktrgq adgfgldilp klkdvkssdn srsllsyivs yylrnfdeda gkeqclfplp
1561 epqdlfqasq mkfedfqkdl rklkkdlkac eveagkvyqv sskehmqpfk enmeqfiiqa
1621 kidqeaeens ltethkcfle ttayffmkpk lgekevspna ffsiwhefss dfkdfwkken
1681 klllqervke aeevcrqkkg kslykikprh dsgikakism kt
```
